# Asynchronous Branch-Parallel Simulation of Detailed Neuron Models

**DOI:** 10.3389/fninf.2019.00054

**Published:** 2019-07-23

**Authors:** Bruno R. C. Magalhães, Thomas Sterling, Michael Hines, Felix Schürmann

**Affiliations:** ^1^Blue Brain Project, École Polytechnique Fédérale de Lausanne (EPFL), Biotech, Geneva, Switzerland; ^2^Department of Intelligent Systems Engineering, CCA Laboratory, Indiana University, Bloomington, IN, United States; ^3^Department of Neuroscience, Yale University, New Haven, CT, United States

**Keywords:** neurosimulation, branch-parallelism, neural networks, asynchronous runtime systems, HPX, ParalleX

## Abstract

Simulations of electrical activity of networks of morphologically detailed neuron models allow for a better understanding of the brain. State-of-the-art simulations describe the dynamics of ionic currents and biochemical processes within branching topological representations of the neurons. Acceleration of such simulation is possible in the weak scaling limit by modeling neurons as indivisible computation units and increasing the computing power. Strong scaling and simulations close to biological time are difficult, yet required, for the study of synaptic plasticity and other use cases requiring simulation of neurons for long periods of time. Current methods rely on parallel Gaussian Elimination, computing triangulation and substitution of many branches simultaneously. Existing limitations are: (a) high heterogeneity of compute time per neuron leads to high computational load imbalance; and (b) difficulty in providing a computation model that fully utilizes the computing resources on distributed multi-core architectures with Single Instruction Multiple Data (SIMD) capabilities. To address these issues, we present a strategy that extracts flow-dependencies between parameters of the ODEs and the algebraic solver of individual neurons. Based on the resulting map of dependencies, we provide three techniques for memory, communication, and computation reorganization that yield a load-balanced distributed asynchronous execution. The new computation model distributes datasets and balances computational workload across a distributed memory space, exposing a tree-based parallelism of neuron topological structure, an embarrassingly parallel execution model of neuron subtrees, and a SIMD acceleration of subtree state updates. The capabilities of our methods are demonstrated on a prototype implementation developed on the core compute kernel of the NEURON scientific application, built on the HPX runtime system for the ParalleX execution model. Our implementation yields an asynchronous distributed and parallel simulation that accelerates single neuron to medium-sized neural networks. Benchmark results display better strong scaling properties, finer-grained parallelism, and lower time to solution compared to the state of the art, on a wide range of distributed multi-core compute architectures.

## 1. Introduction

Interest in the simulation of large neural network activity has been steadily increasing in recent years (Kandel et al., 2013). Experimental advances such as high resolution recording of neurons *in vivo* and *in vitro* have supported quantitative modeling. Biologically inspired simulations of neural circuits present an enormous opportunity for understanding the behavior of the brain. Recent efforts from Markram et al. (2015) presented for the first time a simulation of a morphologically detailed model of a part of the neocortex, simulated in the NEURON simulation environment (Hines and Carnevale, 1997). The simulation was based on the multi-compartment Hodgkin-Huxley (HH) formalism (Hodgkin and Huxley, 1952), a conductance-based model that approximates the current passing through spatially-discretized representations of neuron morphologies. The multi-compartment HH model involves the analytical resolution of stiff, coupled, continuous differential equations on each individual cell, with high variability in time and space scales (Carnevale and Rosenthal, 1992).

The large number of equations involved in such systems leads to computationally costly simulations that may be required to run for long periods of time. A short time to solution is particularly required for the exploration of biological phenomena expressed over long time scales. For example, synaptic weight changes underlying plasticity and learning, on the scale of dozens of minutes to days, in response to continuous millisecond scale neural network activity. Such use cases are studied on networks with a size ranging from two isolated cells (Markram et al., 2012) to thousands or even millions of neurons each with thousands of synaptic connections to other cells (Chindemi, 2018). Since it is not feasible to analytically solve complex HH equations, simulations typically employ standard time-discretized ODE solvers.

Typical efforts to speed-up simulations rely on the parallel and distributed computation of several neurons simultaneously. The theoretical speed-up limit is dictated by the most detailed neuron model in the network, whose state takes the longest to compute. Common approaches follow the Bulk Synchronous Parallel (BSP) execution model, relying on the synchronous parallel execution of several neurons. Multi-core and multi-compute node simulation of branched neuronal morphologies is available in NEURON (Hines and Carnevale, 1997) and other modern neural network simulators. Single Instruction Multiple Data (SIMD or vectorization) optimization of ODE state variables has been implemented in Brian (Goodman and Brette, 2008; Brette and Goodman, 2011), Auryn (Zenke and Gerstner, 2014), and NEST simulators (Gewaltig and Diesmann, 2007) for point neuron representations, and by CoreNeuron (Kumbhar et al., 2016) and Arbor (Klijn et al., 2017) for branched morphologies. Efficient usage of resources is possible if (1) the input includes a dataset large enough to allow enough flexibility to balance neurons across the compute units; and (2) static load balancing is performed beforehand. This has been demonstrated by the Least Processing Time (LPT) algorithm (Korf, 1998), yielding quasi-balanced workload distribution by iteratively assigning neurons to the compute node with the least total computation time. Synaptic communication in distributed executions is typically performed with Message Passing Interface (MPI) (Walker and Dongarra, 1996). A hardware specific and more scalable solution has been presented by Hines et al. on an IBM BlueGene/P using the Deep Computing Messaging Framework (DCMF) runtime (Kumar et al., 2010; Hines et al., 2011), based on immediate selective broadcasts of spikes and a synchronization barrier at the end of every communication step.

Parallelism of individual neuron models has been exploited in a limited way through the NEURON multisplit approach (Hines et al., 2008)—henceforth denominated as previous branch-parallelism efforts or simply multisplit—executed on a single-core, Single Instruction Single Data, distributed compute architecture. The multisplit method implements a parallel computation of neuron branches by converting a given topology into a tree-structure of connecting backbones (linear sequence of unbranched compartments) and leaf subtrees. Tree-based parallelism requires information spawn and reduce throughout connecting subtrees, yielding a limit of parallelism dictated by the slowest subtree to perform a computation step. The method provides a substantial speed-up on the architectures tested at the time, however it lacks support for load-balancing and processing based on SIMD-based acceleration available in modern compute architectures. As an orthogonal effort, spatial decomposition and parallel processing of volumetric regions has been presented by Kozloski et al. for branched neuron models (Kozloski and Wagner, 2011). This approach is most suitable for the simulation of spatially organized and computationally costly elements, as the computation must be large enough to minimize the high communication required—executed at every computation timestep—between neighboring spatial regions in different compute nodes. Moreover, it may yield a high memory overhead and load imbalance due to the duplication (*ghosting*) of compartments in high density regions in networks of detailed neural networks (Magalhães et al., 2016). A complementary acceleration has been proposed by Vooturi et al. (2017), by applying Exact Domain Decomposition to the numerical solver in NEURON (Hines, 1984). The method partitions neuron trees by creating subdomains at the bifurcations with degree greater or equal than two. However, parallelism based on bifurcation points does not properly handle detailed topologies that yield a high disparity of branch lengths, and does not take into account workload imbalance for complex non-HH compartment models.

The various limitations of existing approaches motivate the search for a computation model that accelerates simulations of branched neural networks—independently of the input size—on a wide range of compute architectures, fully utilizing multi-core and vector-based capabilities, exposing finer computation granularity, and running efficiently on single node and distributed networks of compute nodes. Better parallelism of neurons should allow for simulations to run at a runtime closer to real time, and with better usage of the computing resources of modern architectures with hundreds of compute cores and high Single Instruction Multiple Data (SIMD) capabilities, such as Graphical Processing Units (GPUs) or the Intel Knights Landing (Sodani, 2015). Recent efforts such as HPX (Sterling et al., 2014), Charm++ (Kale and Krishnan, 1996), and OmpSs (Duran et al., 2011) provide programming models and runtimes for asynchronous parallelism and heterogeneity. Such tools increase the possibilities of new use cases on the simulations for large-scale HH-based neural simulations, until now restricted to the BSP paradigm and MPI communication model.

**Figure 1:**
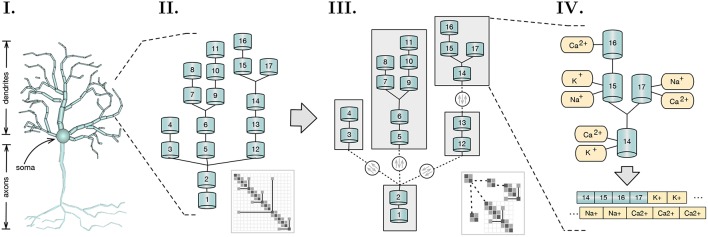
Scope and key concepts of the methods presented. **(I)** A spatial discretization of a neuron into two compartmental trees representing axons and dendrites branching; **(II)** A sample of a dendritic tree and its representative branching matrix structure in thumbnail. Simulation dependency parameters across compartmental connectivity and respective matrix are extracted from these structures; **(III)** A method for load balancing recursively tests for the optimal tree decomposition, based on the computational workload of each possible subtree. Initial compartmental tree is decomposed into a tree of subtrees and distributed across a distributed memory space. Matrix decomposition follows accordingly. Resolution of independent subtrees is computed asynchronously, with three variables dependencies on parent-children connectivity across subtrees (in dashed), synchronized throughout the execution; **(IV)** Subtree memory layouts are reorganized to provide SIMD-acceleration of mechanism (ion channel) state updates and solver resolution.

The work presented introduces novel insights in the field of asynchronous simulation applied to distributed multi-core SIMD-enabled computing platforms. Starting from the work of Hines et al. in branch-parallelism (Hines et al., 2008), we cover the mathematical, computational, and implementational aspects underlying a distributed load-balanced asynchronous parallel simulation of detailed neuron models. The main point of relevance of this work is the acceleration of single neuron simulations. However, as we will later demonstrate, the fine granularity, strong scaling capabilities, and load balancing methods introduced allow as well for a significant acceleration of medium-sized neural networks of up to few thousand neurons. The contributions of the this paper are the following: (a) a formal description of the data dependencies underlying the branching structure of neurons; (b) a method that describes and extracts read-after-write dependencies from the underlying algebraic solver; (c) a load-balancing algorithm that decomposes neuronal representation into a minimal number of subtrees that enable a balanced multi-core execution; (d) a method for the transformation of subtrees into a vector-friendly memory layout that accelerates execution in the SIMD axis; (e) an asynchronous parallel execution model for the resolution of the mathematical dependencies between equations of connecting branches, based on an active producer-consumer execution model of flow dependency variables; and (f) a dynamic finer-grained version of the LPT-based load balancing algorithm for distributed networks, that delegates neuron sections to different compute nodes (henceforth also denominated as localities) in order to balance the workload, while minimizing network communication. An overview of the scope of the research and key conceptual advancements covered in this article are displayed in Figure 1.

The efficiency of the methods presented is demonstrated by a prototypical implementation on the core kernel of the NEURON simulator, with asynchronicity capabilities provided by the HPX runtime system (Sterling et al., 2014) for the ParalleX execution model (Kaiser et al., 2009) on a global address memory space with transactional memory capabilities (Kulkarni et al., 2016). Applied to a network of morphologically-detailed neuron models from Markram et al. (2015), our strategy is shown to provide close to full usage of computing resources (when enough computation is available), finer grained parallelism, and higher multi-threading and SIMD capabilities, validated on three highly heterogeneous neuron models. The benchmarks of individual neurons demonstrate a 1.8x to 10.5x speed-up compared to the reference NEURON multisplit implementation on four distinct compute architectures (Figure 10). Large scale executions yield a speed-up of almost twofold on a network of 128 Cray XE6 compute nodes simulating 4,096 neurons (Figure 12).

## 2. Materials and Methods

### 2.1. Numerical Resolution

Our reference implementation follows the explicit resolution using staggered timestepping of the NEURON scientific simulator. In our model, neurons are described by a tree topology of resistors, with capacitors and non-linear resistive current flows at each node connected to ground. Each of the complex nodes is also referred to as a compartment. The current passing through the membrane of a single compartment *n* with difference in potential (across its membrane) *V*_*n*_ can be described as:

(1) $$\begin{array}{r} I_{n}\left(t\right)=C_{n}\frac{dV_{n}}{dt}+\underset{i}{\sum }g_{i}x_{i}\left(V_{n}-E_{i}\right) \end{array}$$

where *g*_*i*_, and *E*_*i*_ describe the conductance and reversal potential of the different current channels in the system, respectively. *I*_*n*_(*t*) is the synaptic current of an injected current stimuli, if any. When applicable, *x*_*i*_ is the voltage-dependent variable(s) defining the opening and/or closing of the ion channels, and typically described as a first-order voltage-dependent differential equation. For brevity, the formulation of the ionic currents from the opening variables are omitted.

A branched representation of a neuron allows for larger spatial resolution, by adding the neighboring compartments' contributions according to the neuronal cable theory for multiple compartments (Niebur, 2008). However, ion channels and other biological mechanisms are normally distributed along the neuron topology in an heterogeneous way. Thus, the computational workload of individual compartments may vary extensively. Following the compartment numbering convention used by NEURON (Hines et al., 2008), the tree of compartments is numbered using a Depth-First Search scheme from root to leaves, ensuring that the index of a parent compartment is larger than all its children and smaller than its parent. Such a matrix guarantees that the matrix is symmetric and in each row *i* there exists a single non-zero element with columns index *j* such that *j* < *i*, i.e., a single parent compartment per branch. Branched neuron trees include the terms defining the currents derived from the parent and children branches, leading to the final formulation:

(2) $$\begin{array}{l} I_{n}\left(t\right)=C_{n}\frac{dV_{n}}{dt}\;+\;\underset{i}{\sum }g_{i}x_{i}\left(V_{n}-E_{i}\right)\;+\underset{c:p\left(c\right)=n}{\sum }\frac{V_{c}-V_{n}}{r_{c}}\\ \;-\frac{V_{n}-V_{p\left(n\right)}}{r_{p\left(n\right)}} \end{array}$$

where *p*(*c*):ℕ → ℕ returns the index of the parent compartment of a given compartment *c* and *r*_*c*_ is the resistance of the connectivity to neighboring compartments, if any. Due to having no analytic solution, numerical methods are employed with a problem-optimized solver used for the resolution of the system of equations.

**Figure 2:**
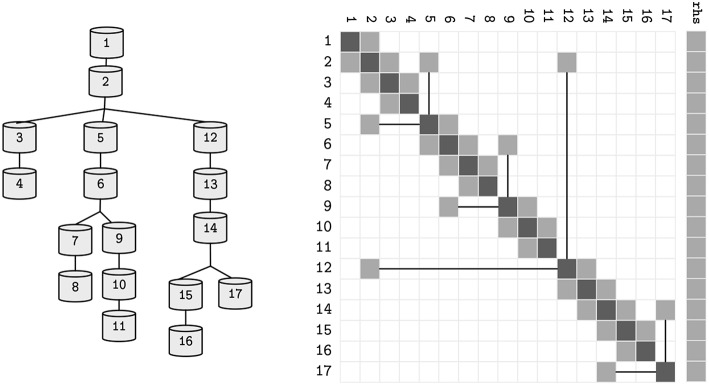
The topological structure of a sample neuron, and its representative sparse tridiagonal dendritic tree representation. Lower and upper diagonals include parent and children contributions to the current function (*a* and *b* in Equation 3). Main diagonal (*d*) includes the changes to the compartment voltage *dV*/*dt* induced by the capacitance and mechanisms. Remaining terms are included in the right hand side (*rhs*) vector. Straight lines display parent-to-children connections between compartments with non-sequential indices (referenced by *p*).

NEURON's algebraic solver (Hines, 1984) describes each neuron as a sparse-tridiagonal matrix that represents the voltage in a compartment as a function of the main diagonal of the matrix, the contributions from parents and children on the upper and lower diagonals, respectively, and the mechanism contributions to the voltage on the right hand side of the matrix-vector multiplication. Upon the computation of all mechanism contributions, all compartments must solve:

(3) $$\begin{array}{r} b_{p\left(n\right)}V_{p\left(n\right)}+d_{n}V_{n}+\underset{c:\;p\left(c\right)=n}{\sum }a_{c}V_{c}=rhs_{n} \end{array}$$

where *d, a, b* ∈ ℝ^*N*^ are the coefficients of the voltage contribution for the compartment, its children and parent compartment, on a neuron with *N* compartments—refer to Figure 2 for a sample neuron and its sparse tridiagonal matrix representation. As previously, *V* is the difference in potential across the compartment's membrane and *p*(*n*) returns the parent index of a compartment *n*. The right hand side term *rhs* holds the remaining terms. Given a tree with the aforementioned terms updated, the final solution of the system (*rhs*) can be computed by a problem-specific implementation of the Gaussian Elimination, that only modifies the *d* and *rhs* vectors, as *a* and *b* are constant.

**Figure 3:**
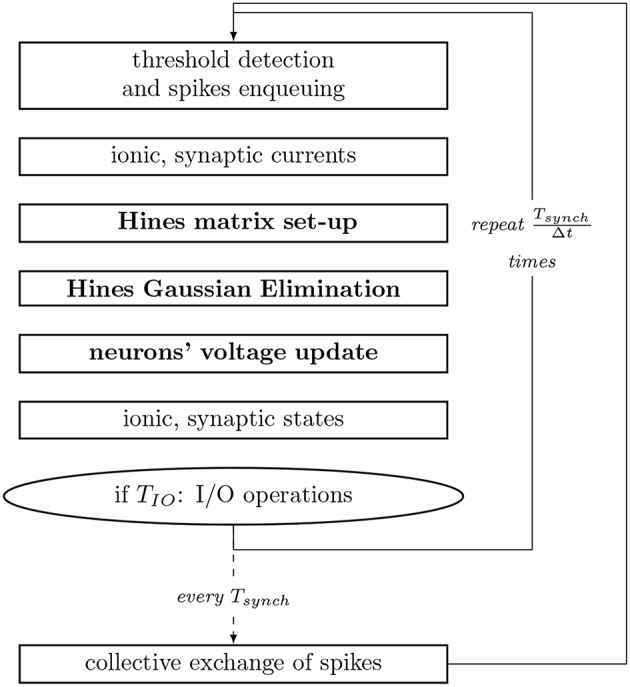
Workflow of a compute step of the core computation of NEURON used as reference implementation. *T*_*synch*_ and *T*_*IO*_ are the communication and IO step intervals, respectively. Processes with inter-compartmental data dependencies are presented in bold. Single compute node simulations exclude the step for collective exchange of spikes.

For the numerical resolution of state updates, NEURON performs a spatial discretization of the neuronal morphology and interpolates the solution only at consecutive discrete time intervals. The coupling between the main current equation and the set of ionic states is resolved in a staggered fashion, in interleaved half timesteps. Moreover, it assumes the spatial discretization to be small enough, so that the second order correctness only implies that the value at a node (center of the compartment) is the average value in the compartment, allowing compartments to be interpolated throughout time only, and reducing the spatio-temporal dimensionality of the system. Subsequently, the state of the axonic branches are assumed to be constant throughout the execution, therefore simulating only soma and dendrite sections. Thus, upon an action potential (spike) of a neuron, the synaptic propagation delay (time dependency) between two neurons includes the current propagation from the soma (or axon initial segment where the action potential has a constant velocity and can be represented as a triggered event) to the bouton in the axon, plus the time required for the electro-chemical reaction at the synapse. This interval varies extensively across pairs of neurons. The shortest propagation delay in a neural network—of circa 0.1 ms in our model—is used as the synchronization and communication step size for the exchange of synapses to be delivered within the next interval. The computation step time is defined to be short enough to capture the resolution of the fastest mechanism, and is typically set to 0.025 ms. For completeness, Figure 3 summarizes the workflow of a computation timestep, and provides the runtime of individual processes.

### 2.2. HPX Implementation

Our methods were implemented on the core kernel of the NEURON simulator, available as open source (Blue Brain Project, 2015; Kumbhar et al., 2019). Communication, memory handling and threading protocols were replaced by primitives provided by the HPX runtime system for ParalleX. HPX allows for a platform-agnostic representation of data structures and distributed computation on a Global Memory Address Space (GAS). Data objects are not locally addressable on a machine, but defined by a global reference instead, thus leading to a high level abstraction of memory allocations across the network. The global memory address space and transactional memory capabilities available on the HPX runtime system allow for procedure calls, synchronization objects (mutual exclusion, semaphores, thread gates), and memory allocating functions, to be performed asynchronously and remotely as if they were local, with *futures* guiding their state. Therefore, the implementation on single and multiple compute nodes of the methods presented are indistinguishable, and data structures are automatically represented and transparently accessible across any network configuration.

The results presented next rely on the efficient implementation of HPX control objects across neuron subtrees (or subsets of the initial neuron topology), built on a distributed memory space. The most relevant implementation features are:

- Subtrees are allocated on the Global Memory Address Space (GAS), with a physical allocation on the compute node provided by the load-balancing method detailed later in the manuscript. Each GAS address is unique and its pointed object can be transparently accessed from any compute node. The GAS addresses of connecting parent-children subtrees and synaptically-connected neurons are shared at the onset of the execution;
- Synaptic deliveries are performed with a remote procedure call to the address of the target (post-synaptic) subtree, with spiking time as argument;
- Synchronization of neurons time advancement is enforced with a communication barrier at equidistant time frames, equivalent to the smallest synaptic delay in the network;
- Computation flow on each neuron is guided by threads attached to the shared placeholders between connecting subtrees, that go dormant and active when waiting for a placeholder or upon a placeholder value update. The HPX thread scheduler handles the scheduling of compute resources to the queued compute kernels;
- Shared placeholders for dependency variables among connecting subtrees are built on top of asynchronous calls to *set* and *get* operations, supported by a *future* for probing of state. A thread gate (or *and gate*) underlies the implementation of each placeholder, with an initial counter set to the number of contributions that must be set before execution is allowed to continue. There is one contribution to be set for parent-to-children dependencies, and a number of contributions equal to the number of children in the converse direction;
- The distributed execution on the GAS is based on remote procedure calls, that initiates a lightweight thread on the appropriate compute node, or places it in a queue of dormant threads to be dynamically allocated to idle compute resources throughout the execution. Upon the execution of the method at a remote location, an asynchronous call is sent back to the request initiator with the return value. The runtime system handles communication, execution and callbacks for the remote procedure calls;

Finally, to allow for efficient point to point communication, selective broadcasts, and remote direct access memory, we use specialized Infiniband network hardware, interfaced via the photon API library (Kissel and Swany, 2016).

## 3. Results

### 3.1. Dependency Parameters

**Figure 4:**
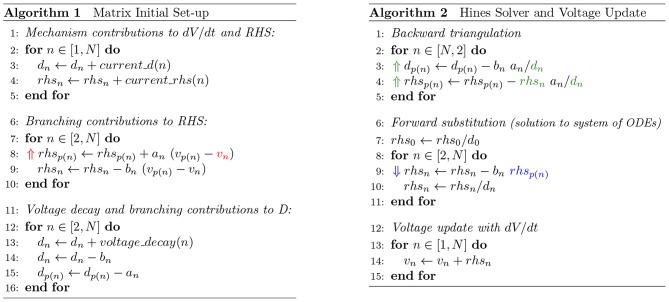
Algorithms of the initial matrix values set-up (left) and Gaussian Elimination and voltage update methods (right) of a neuron discretized by *N* compartments. Data-dependency variables are emphasized in colored text, with direction of arrow in same color pointing up if children-to-parent flow dependency, or down otherwise. Variables notation follow Equation (3).

We extracted the mathematical dependencies from the numerical resolution of the electrical activity in the compartmental tree. The dependencies are retrieved from the matrix set-up and Gaussian Elimination operations, presented in Figure 4. Parameters that are required to be communicated between connecting compartments are color-coded. The direction of the parameter flow dependencies is indicated by the direction of the arrow in the same color—top-down for parent-to-children, bottom-up for children-to-parent. The read-after-write dependencies are:

1. *Branching contributions to RHS*: the set-up of the Right Hand Side requires the contribution of the difference in potential between connecting compartments (*v*_*p*(*n*)_−*v*_*n*_) and their constant branching contributions *a*_*n*_ and *b*_*n*_. An updated voltage from children to parent (⇑*v*_*n*_) allows for the computation of the *rhs* vector set-up, yielding a children-to-parent flow dependency;
2. *Backward triangulation*: a children-to-parent flow dependency allows for the completion of the Backward Triangulation step by providing the children compartment contributions to their parents' diagonal and right hand side values (⇑*d*_*n*_, *rhs*_*n*_);
3. *Forward substitution*: *rhs* values are required to be modified in the root-to-leaves direction, in order to compute the final value of children's *rhs*, leading to a parent-to-children flow dependency [⇓*rhs*_*p*(*n*)_]. The outcome of the substitution step is the updated *rhs* for the current step, i.e., the voltage values;

For subtrees with a single connection point to a parent node, the back-triangulation is complete, i.e., triangulation at its parent subtree can be initiated immediately after. For subtrees with two connection points, triangulation stalls until the triangulation on both children branches is completed. Substitution follows a converse logic, being optimally computed for leaf (single connection point) subtrees, and requiring substitution at parent subtrees to be completed beforehand if not a leaf subtree.

Mechanism state update of compartments (*current* and *state* functions in Figure 3) can be performed independently, as long as the previous dependency variables are resolved. Thus, the finest granularity of parallelism (or the smallest compute task) can now be modeled at the compartment level. More importantly, granularity of tasks can be increased and decreased by clustering connecting compartments. With that in mind, the following section provides a clustering method that takes advantage of this property to perform load-balancing on multi-core execution units.

### 3.2. Neuron Tree Decomposition and Parallel Execution

The problem of scaling the model of a neuron efficiently across any number of compute units with vectorization (SIMD) capabilities is two-fold: at first, there should be a *large enough* number of compute tasks to provide enough flexibility in the distribution of tasks. The rationale is that a high number of tasks allows for a better balancing of the total workload (sum of task runtimes) across processors. Secondly, the number of total tasks should ideally be as small as possible, so that vectorization can be maximized inside each task and threading (de)allocation overhead is minimized. This leads to a competitive trade-off between multi-core and vector acceleration.

**Figure 5:**
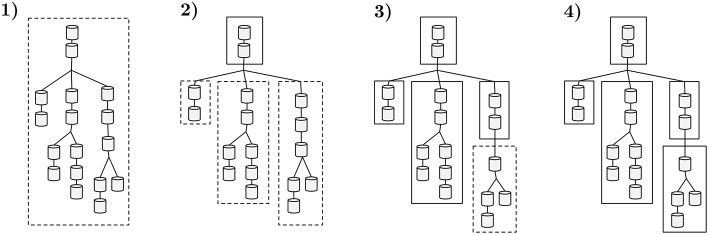
A sample workflow of the algorithm that decomposes neuron morphologies into a tree of subtrees. Dashed contours display groups of compartments being tested against the maximum computational complexity threshold. Straight contours represent a set of compartments with a total computational complexity below the threshold, clustered into a subtree. **(1)** The total runtime of the initial tree is computed and compared with the complexity threshold. **(2)** The previous cluster exceeds the allowed computational complexity threshold. The following sequence of non-bifurcated compartments that yields smaller complexity (top two compartments) is clustered into a subtree. The connecting three branches are tested. **(3)** Both left and center sections are benchmarked and their execution time is below the threshold, thus becoming two independent subtrees. The same threshold test for the right region fails. The first two compartments in the right region yield less runtime than the complexity threshold and are clustered into a subtree. **(4)** The remaining compartments on the right branch of the tree comply with the complexity test and are clustered, leading to the final data representation.

To fine-tune the data representation to the host compute architecture, our strategy takes advantage of the flexibility in granularity presented in the previous section. The algorithm responsible for loading data structures into memory, recursively traverses the neuron tree and clusters connecting compartments that yield a computational workload as close as possible to a given threshold. The cluster is a subtree of compartments whose computational complexity is provided by the runtime of all compartments it contains. Remaining compartments, not included in the previous subtree, will recursively be clustered using the same maximum-threshold testing algorithm, until all compartments are traversed. Bifurcations require independent tests on each branch. Thus, if a branch connects to several children, then all branches in the lower level are either in the same subtree as the parent, or on independent subtrees. This rule avoids having subtrees connected to compartments that are neither root or leaf in other subtrees, so that synchronization of computation is not required except at terminal compartment connections. The maximum computation time assigned to a subtree of a given neuron *n* is scaled by a constant *k* and provided by:

(4) $$\begin{array}{r} max\;work_{subtree}\left(n\right)=k\frac{runtime_{n}}{cores\;count} \end{array}$$

an approach similar to NEURON's multisplit which can be interpreted as *each subtree must yield a max computational workload to fit at most* 1/*k* *subtrees per compute core*. An analogous interpretation is that the initial neuron tree is decomposed in several subtrees, each guaranteed to have a runtime of at most *runtime*_*n*_/*cores count* scaled to a factor *k*—where the constant *k* provides flexibility in the number of subtrees generated, essential for load balance of computation across compute cores. An illustrative example of the application of the clustering algorithm to a single neuron is presented in Figure 5. Two cases may lead to a subtree with an assigned workload which does not approximate (Equation 4): a linear sequence of compartments of small computational complexity that requires a bifurcation into multiple subtrees; or a leaf subtree of the topology that does not aggregate enough complexity.

**Figure 6:**
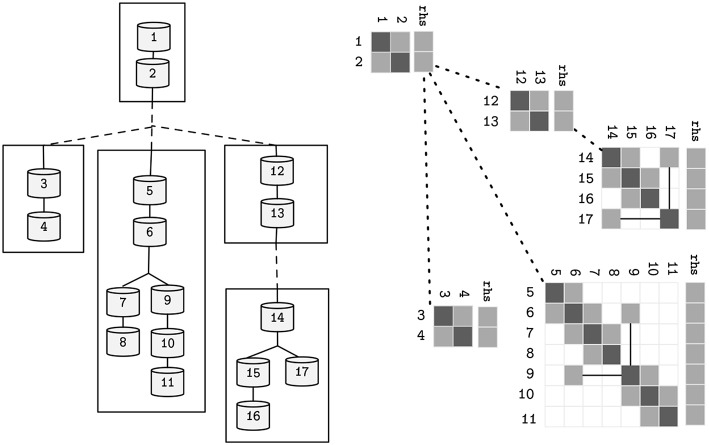
Alternative representations of the neuron model presented in Figure 2, after the decomposition into subtrees pictured in Figure 5. Straight lines represent parent-children data-dependencies within the same subtree. Dashed lines represent data dependencies to different subtrees.

Following the partitioning algorithm, parallelism of subtrees is possible, requiring synchronization of the connecting flow dependencies at every computation step. Due to the recursive tree-traversal nature of the algorithm, the final representation of the neuron is a tree of subtrees, where each subtree is a tree of compartments by itself. Analogously, the initial solver problem is now decomposed into several smaller solver problems with a data dependency between only the root and leaf rows in different subtrees—refer to Figure 6 for an illustrative sample of a neuron and its linear solver structure after the clustering method. This property allows for a vector-based acceleration of individual subtrees, as will be covered in the following section.

### 3.3. Vector-Based Acceleration

**Figure 7:**
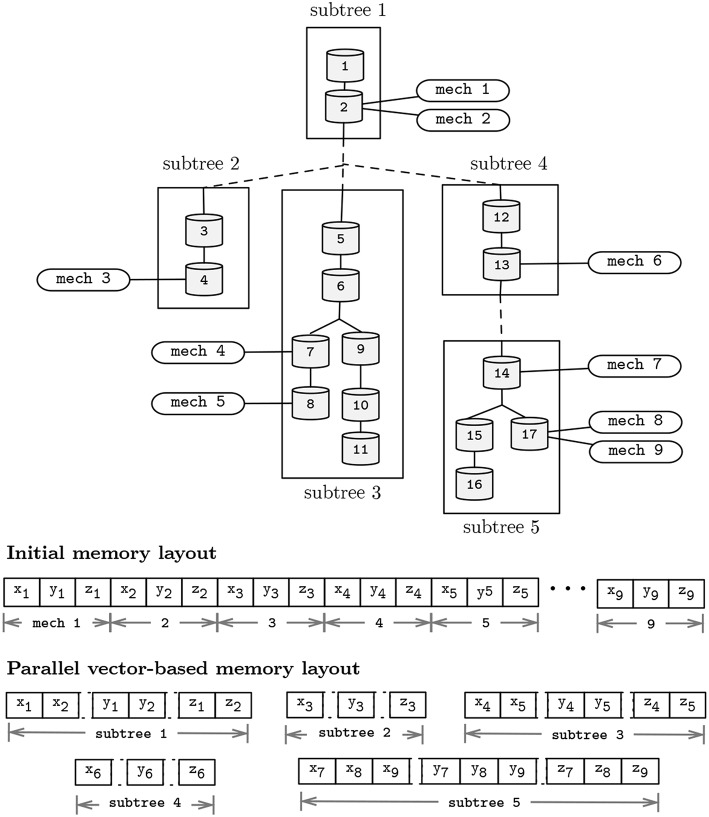
Representation of a neuron topological structure after clustering into 5 subtrees (top); and memory layouts for the pre- and post-subtree clustering phases presented in Figure 5 (bottom). Morphology includes 17 compartments, and 9 instances (mech 1–9) of a mechanism type with state variables *x*, *y*, and *z*. Distinct mechanism types and solver parameters *a*, *b*, *p*, *d*, *v*, and *rhs* were omitted for simplicity.

Similarity in the computation of the mechanisms and compartments update function allow for an acceleration in the SIMD-axis by performing vectorized computation of state variables. Vectorization can be enabled in two distinct core computations: (1) on the execution of the Gaussian Elimination method, by performing simultaneous (memory-aligned) read operations of *a*, *b*, and *p*, and updating the variables *d*, *v*, and *rhs*; and (2) vectorization can be achieved by performing simultaneous computation of instances of the same mechanism types, placed in different compartments, holding different states, yet defined by similar state-update functions. To enable vectorization, the memory layout for individual subtrees is serialized and realigned on a SIMD-friendly layout, after the subtrees decomposition algorithm presented. For completeness, Figure 7 displays the post-vectorization memory layout for the morphological representation studied previously.

The computation workload allocated to each task (subtree) during load balancing is measured as the runtime of the subtree in the vectorized memory layout. In practice, a test for the computational complexity of a subtree requires the tentative set of compartments to be merged and SIMD memory-arranged. As high disparity in workload across subtrees may occur, ideal core workload balancing is not guaranteed. An asynchronous execution model mitigates this issue by dividing the subtree stepping workflow in smaller kernels and dynamically running available kernels as soon as dependency variables are resolved. This procedure is detailed next.

### 3.4. Asynchronous Execution of State Updates

To handle the disparity in computation times across subtrees and to fully utilize compute resources, an asynchronous producer-consumer execution model was implemented. Flow dependencies can be resolved by actively exchanging information between connecting subtrees, providing parent and bottom subtree contributions required for the completion of the Gaussian Elimination step. Execution is driven by shared placeholders across connecting subtrees, that stall the execution and resume it when dependency variables are updated for the current step, as detailed previously in section 2.2.

**Figure 8:**
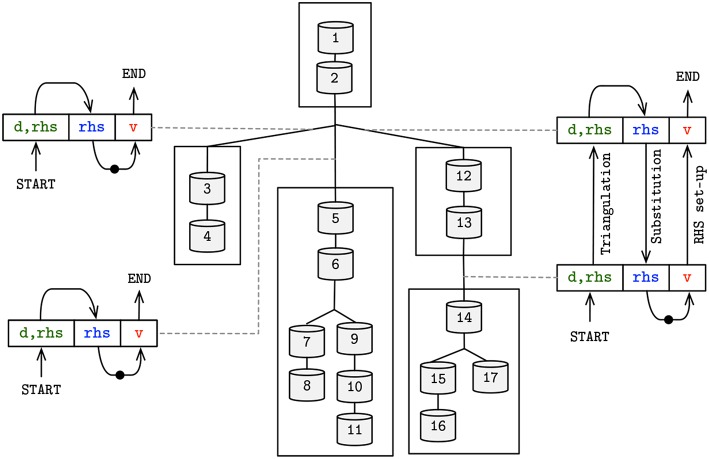
Schematic overview of the asynchronous producer-consumer model, displaying the shared placeholders between subtrees—colored and matching (Figure 4). Arrow head (tail) on placeholders represents a set (get) operation by the producer (consumer) of the placeholder's value. Dots in arrow line comprise the update of mechanism states, leading to an updated voltage value to be used in the following iteration.

Figure 8 provides the diagram for the resolution of flow dependencies based on the three placeholders demonstrated previously, in line with the algorithm displayed in Figure 4. The combination of SIMD-enabled compute tasks, placed in and out of context of compute cores throughout execution, allows for an asynchronous execution model and the full utilization of computing resources on a single compute node.

### 3.5. Distributed Load Balancing

The extension of the methods presented to a network of compute nodes relies on a load balancing algorithm that aims to equalize the workload across the network, while minimizing inter-node communication. To that extent, the previously mentioned vector vs. multicore efficiency trade-off is extended with a communication optimization on distributed localities: the framework must now deliver enough balanced SIMD-tasks that utilize all compute cores across the network, while minimizing the inter-node communication from the placeholders connecting neuron sections held on different localities.

The load balancing algorithm implemented follows a distributed implementation of the Least Processing Time algorithm (Korf, 1998). To allow finer-granularity in the load balancing, the LPT is applied to groups of connected neuron subtrees instead of whole neurons. The method relies on the active update of a shared table holding the total computational time assigned to each locality so far. At the onset of the execution, neurons are loaded across the network, assigned a weight based on a computational workload (measured as the mean runtime of 10 sample 100 ms simulations of each subtree), serialized and finally communicated to the least busy compute node. As a reminder, the workload of individual subtrees remains quasi-constant throughout the execution, thus justifying (1) static load balancing, once and at the onset of execution, and (2) the usage of a computationally-heavier yet very accurate metric of computational workload (weight) based on simulated runtime and not on predictive methods. In most cases, only whole neurons are moved to other localities. This rationale allows connecting subtrees to be placed within the same memory region, and reducing inter-node communication—required once at every synaptic delay between connecting neurons, and three times per step for connecting subtrees. However, terminal (leaf) subtree groups of a neuron may be assigned to a different locality if the load imbalance caused by a whole-cell placement in a single memory region exceeds the locality runtime threshold. To avoid communication delays caused by more than two computed nodes involved in any resolution of a single neuron, only terminal sections of neurons are delegated to a remote compute node. This rationale avoids transitive communications: in practice, dividing a single neuron across three localities 1..3 may yield a communication pattern of 1 → 3 and 2 → 3 for two unconnected arborizations 2 and 3, but not 1 → 2 → 3. The maximum computation threshold assigned to each compute node is provided by the mean runtime of the dataset per locality—computed at the onset of the execution—with a tolerance value of about 10% that, when exceeded, signals the delegation of the remaining arborization of a neuron to a remote memory locality.

### 3.6. Benchmark

**Figure 9:**
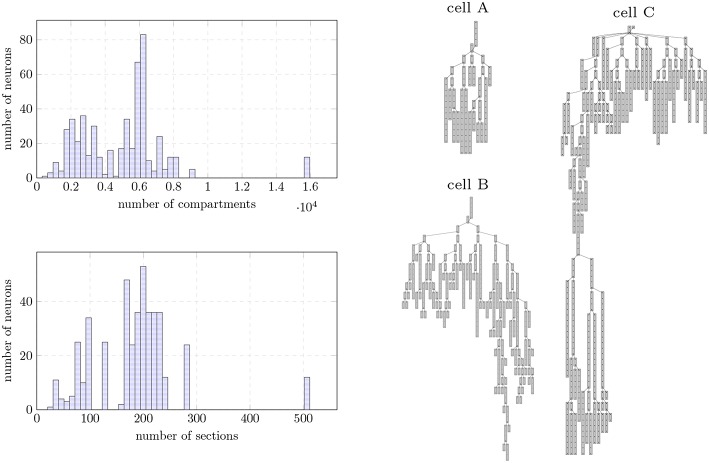
**(Left)** Morphological structure of the Layer 5 neurons extracted from the digital reconstruction of the rodent neocortex used as input data. Data provided as a histogram (50 bins) in terms of distribution of number compartments (top) and number of sections (unbranched cables of a neuron, bottom). **(Right)** Dendritic compartmental trees of the three individual neurons used as input dataset on single node benchmarks.

Our test bench simulates the biological activity of a digital reconstruction of a morphologically-detailed neural network extracted from the model of Markram et al. (2015). The reconstruction model underlies the research of synaptic plasticity and learning from The Blue Brain Project (Chindemi, 2018), requiring a simulation of dozens of minutes to several days to be expressed. Input neurons were extracted from the layer 5 of rodent brain neocortex. Neuron models include 23 distinct biological mechanism types modeled by 44 ODEs, and highly heterogeneous neuron topologies—see Figure 9 for the distribution of compartments and branches across the dataset. Each neuron requires 4–10 MB of memory for the storage of the topological structure, linear solver, mechanisms states, and dynamic data containers for synaptic events.

Execution times on a single compute node were measured on four distinct compute architectures: (1) an Intel Sandy Bridge E5-2670 with 16 cores at 2.6 GHz, with and AVX capabilities (256-bit floating point vector operations), and 128 GB of RAM; (2) an Intel Knights Landing (KNL) with 64 cores at 1.3 GHz, 96 GB of RAM, and AVX-512 (512-bits register file width); (3) a Cray XE6 with 2x AMD Opteron 6380 with 16 cores at 2.5 GHz each, 64 GB of RAM and 256-bit floating point units; and (4) an Intel Xeon Gold 6140 with 18-core at 2.3 GHz with AVX-512, turbo-boost up to 3.7 GHz, and 98 GB RAM. State values are stored at double floating-point precision, leading to a maximum SIMD speed-up of 4 and 8 simultaneous operations for 256- and 512-bit register file width, respectively.

The optimal value of the constant *k* defining the maximum computational complexity per subtree was computed with a grid search between 0.1 and 2 with a step of 0.2, a method commonly used in problems of the same domain (Hines et al., 2008). The optimal value depends on the number of active cores available at runtime, and was measured at approximately *k* = 0.8 for 2 cores, *k* = 1 for 4 cores, *k* = 1.5 for 8 cores, *k* = 1.8 for 16 cores, and *k* = 2 for more than 16 cores. A deviation of circa 20% over the aforementioned values is possible, as it depends on the input morphology and architecture.

For brevity, in the following benchmarks we will refer to our implementation as neurox, as in NEURON on HPX. Moreover, the initial load balancing and memory layout realignment processes take ~1–2 s of execution time and are excluded from the analysis, as they are considered negligible in the overall execution.

#### 3.6.1. Reference Branch-Parallel Implementation

**Figure 10:**
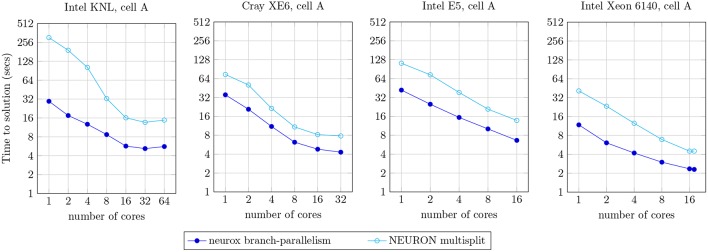
Strong scaling plot for the simulation of one second of electrical activity of the neuron A, illustrated in Figure 9. Benchmark results presented for the NEURON multisplit approach, and our methods (neurox). Hardware specifications: Intel KNL with 64 cores at 1.3 GHz and AVX-512; Cray XE6 compute node with 2× AMD Opteron 6380 with 16 cores at 2.5 GHz; Intel E5 with 16 cores at 2.6 GHz and AVX2; and Intel Xeon Gold 6140 with 18-core at 2.3 GHz with AVX-512.

We compared our methods against the reference branch-parallel implementation in the NEURON multisplit (Hines et al., 2008). Our test bench measures the runtime of the simulation of one second of the electrical activity of the cell A (illustrated in Figure 9), on the four compute architectures mentioned previously. The benchmark results are presented in Figure 10. The speed-up achieved was of approximately: 10.5x for the single-core execution, down to 2.64x on the 64-core execution on the Intel KNL; 2.2x–1.8x for single- to 32-core execution on the dual-AMD Opteron; 3.2x–1.8x for single- to 32-core on the Intel E5; and 3.5x–1.9x on the single- and 18-core implementation on the Intel Xeon 6140. As expected, better acceleration is achieved by the Intel Xeon and KNL architectures, mostly noticeable for a small number of cores, as the register file width is twice the amount of the dual-Opteron and E5.

As an important remark, some of the single-core runtimes presented (particularly the KNL) exceed the theoretical limit of SIMD speed-up of 4x or 8x. This efficiency increase is due to the structure-of-arrays data layout in memory allowing better memory access compared to the non-SIMD array-of-structures, allowing an extra speed-up on top of the SIMD instruction-level parallelism.

#### 3.6.2. Scaling of Single Neurons

**Figure 11:**
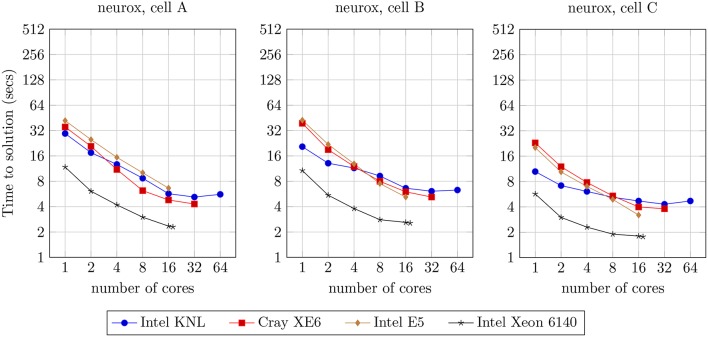
Strong scaling benchmark for the simulation of one second of electrical activity of three different models of single neurons illustrated in Figure 9, benchmarked on four compute architectures: Intel KNL with 64 cores at 1.3 GHz and AVX-512; Cray XE6 compute node with 2× AMD Opteron 6380 with 16 cores at 2.5 GHz; Intel E5 with 16 cores at 2.6 GHz and AVX2; and Intel Xeon Gold 6140 with 18-core at 2.3 GHz with AVX-512.

We analyzed the strong scaling properties of our methods, simulating one second of electrical activity of three neuron morphologies characterized by different levels of width and depth of branching, illustrated in Figure 9. The benchmark results are presented in Figure 11. This analysis provides the theoretical limit of acceleration for single-neuron execution, as a basis for the study of the acceleration for the larger networks of neurons that will follow. As a first remark, it is noticeable that the single-core runtime of individual neurons is not related to the branching density of each neuron model. This is due to the computational workload of each neuron being not derived from the branching-related computation, but mostly from the computational workload attached to ionic current and state updates (Figure 3).

On the comparison of single-core with maximum-core execution runtimes on four distinct compute architectures, the benchmarks demonstrate a speed-up of: 5.3x, 3.3x, and 2.3x for cells A, B, and C, respectively, on the Intel KNL; 8.2x, 7.5x, and 6.0x on the dual AMD Opteron; 6.3x, 8.2x, and 6.3x on the Intel E5; and 5.1x, 4.2x and 3.2x on the Intel Xeon 6140.

Almost linear strong scaling on up to 8 threads on single-core compute units with Single Instruction Single Data processing has been previously demonstrated by the NEURON multisplit approach (Hines et al., 2008). Similarly, in our methods, ideal scaling is not visible and an acceleration beyond 16 threads per neuron is strenuous. This limitation has already been studied in the multisplit method by Hines et al. (2008), and is unrelated to the implementation but due to the nature of the problem instead. Subtrees are quasi-balanced in terms of workload, yet stochastic temporal events such as user-defined current injections and synaptic activity account for extra computation that can not be accounted for by the subtree clustering algorithm, leading to an unpredictable imbalance at certain time intervals. More importantly, the tree structure of the dataset limits the parallelism exposed when computation is concentrated at higher levels of the neuron tree, such as during Gaussian Elimination. This property is noticeable when comparing the runtimes of the three cells A–C with increasing branching depth, where an increase of the cell depth leads to a reduction in the strong scaling capabilities of our method.

Finally, the reduction of the speed-up with the increase of compute cores is also related to the aforementioned trade-off between SIMD parallelism (maximized for single core execution) and vector-based parallelism: to fully utilize all available cores, smaller SIMD data structures are created, causing a loss on the exposed vector acceleration, i.e., register file width is not fully utilized. As a smaller factor of performance loss, an extra overhead is added by threading (de)allocation as we increase the number of active cores.

#### 3.6.3. Scaling of Networks of Neurons

An analysis of scaling of our implementation on a larger dataset was executed on a network of 128 Cray XE6 compute nodes, performing a simulation of one second of electrical activity on the same neocortical model. The benchmark presents the scaling properties of our branch-parallel implementation, similar to the previous section, and compares it against the non branch-parallel counterpart, that simulates neurons as indivisible units.

**Figure 12:**
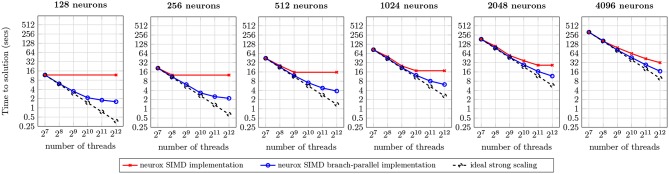
Benchmark of the simulation of one second of electrical activity of Layer 5 neurons, on a Cray XE6 cluster with 128 compute nodes. Each node contains two AMD Opteron 6380 with 16 cores at 2.5 GHz each with 256-bit register file width. Ideal strong scaling assumes complete overlap of computation and communication.

The execution runtime for an increasing number of neurons is presented in Figure 12. The first (leftmost) benchmark illustrates a strong scaling analysis of one neuron per compute node, similar to the previous (single node, single locality) benchmarks. The results demonstrate the preservation of strong scaling properties between the single and 128 compute nodes use cases. Similar benchmarks were performed for 16, 32, and 64 compute nodes—omitted for brevity—and provide identical results. A weak scaling analysis follows by increasing the workload from 128 to 4,096 neurons (following the benchmarks from left to right), in the same hardware and number of compute units. Results suggest that an increase of the input dataset per compute node approximates the runtime to the ideal strong scaling limit. This is due to an overlap of computation and communication across different neurons subtrees, leading to a better usage of compute resources.

The maximum speed-ups measured were of 7.4x for 128 neurons, 5.8x for 256 neurons, 4.2x for 512 neurons, 2.8x for 1,024 neurons, 2.3x for 2,048 neurons, and 1.9x for 4,096 neurons. For the 128–2,048 neuron datasets, most of the speed-ups are due to the saturation of threads with compute tasks on the branch-parallel execution, but not on the non-branched execution (due to an insufficient number of neurons). However, in the scenarios where both implementations fully utilize their compute units, a significant speed-up is noticeable. This property is more prominent on the largest dataset tested, where the dataset yields one neuron per thread, thus providing enough tasks to fully occupy all computing resources on both implementations, and speed-up of almost twofold is visible. This speed-up is due to the finer-grained parallelism and load balancing exposed by our methods, allowing better dynamic allocation of tasks to threads, thus leading to an overall reduction in runtime.

## Discussion

This paper presented an algorithm and an implementation that extract numerical dependencies at the topological level of neurons, and accelerate the simulation of morphologically-detailed neuron models. We detailed the method for the numerical resolution of our problem, and showed that (1) the activity of the electrical current at the level of neuron topological trees depends on three parameters of the numerical solver that include current contributions between connecting tree sections; (2) the previous dependency allows for the grouping of connecting compartments into subtrees, where each subtree is a subset of the initial problem, and the set of substrees holds the same data representation as the initial neuron; and (3) subtrees can be grouped into a tree of subtrees—holding the previous cover and distinct set properties—and allocated to any locality on the network in order to allow for accurate distributed load balancing.

Our analysis showed that a numerical resolution with full usage of compute resources on a distributed network of compute nodes is possible at three layers of parallelism: (1) at the level of compute nodes, a load balancing method delegates sections of neuron arborizations to localities at the onset of execution; (2) at the compute node level, load balancing follows analogously with the clustering of compartments into subtrees, allowing a multi-core acceleration by dynamically delegating subtrees to compute cores throughout the execution; and (3) at the core level, where SIMD-based acceleration of state variable updates and numerical solver acceleration is possible by realigning the memory layout of each subtree.

The methods were implemented on the compute kernel of the NEURON scientific application, yielding an asynchronous simulator on a global memory address space, with synchronization and threading supported by the HPX runtime system. The benchmark results compared our methods with the reference branch-parallelism method in the NEURON simulator, yielding a speed-up of up to 10.5x on an Intel Knights Landing with 64 cores, 2.2x on a Cray XE6 compute node with 2x 16-core AMD Opteron, 3.2x on an 16-core Intel E5, and 3.5x on a 18-core Intel Xeon 6140. A following benchmark studied the efficiency of our methods on three highly heterogeneous neuron models, displaying good strong scaling properties on up to 16 cores, and a dependency of the parallelism efficiency on the depth of the neuron tree.

We extended our methods to larger neuron networks, and assessed their scaling properties on a network of 128 Cray XE6 compute nodes simulating up to 4,096 neurons. Our implementation was shown to deliver a speed-up of 7.4x for small datasets, and 1.9x when full occupancy of compute resources was guaranteed. Moreover, it displayed a good preservation of its strong scaling properties, with almost ideal scaling on the largest dataset tested.

The preservation of the scaling properties—independently of the network size and compute cores per neuron—combined with the added capability of generating a varying number of compute tasks allocated in a balanced way across all localities, allows our strategy to be fine-tuned to a wide range of distributed architectures and inputs.

Finer-grained parallelism is a requirement for succeeding in efficiently leveraging the compute capabilities of modern compute architectures. The combination of the methods presented allows for the detachment of the problem representation from the hardware specifications, and introduces—to our knowledge—the first implementation of a distributed, multi-core, SIMD-enabled simulator that adapts the data memory layout to the host architecture on a distributed network of compute nodes, opening the door for new possibilities in approximating the runtime simulation of morphologically detailed neuron models to real time. Our methods provide insights for the design of future simulators across a wide range of scientific domains, driven by large heterogeneous datasets and compute architectures.

As a final remark, a further acceleration of the simulation can be achieved by (1) graph-parallelism extracted from the dependencies between ODEs driving individual neurons or subtrees, and (2) the removal of the synaptic exchange synchronization barrier on distributed networks, with a cache-efficient stepping of neurons based on the synaptic delay of their pre-synaptic counterparts. These two properties are part of ongoing work and will be included in future manuscripts.

## Data Availability

The source code of the neuron implementation presented can be found in the Blue Brain's neurox repository (Magalhães, Bruno RC and Blue Brain Project, 2017). The reference CoreNEURON implementation can be found in the Blue Brain CoreNEURON's repository (Blue Brain Project, 2015). The datasets for this study will be made available by the authors, without undue reservation, to any qualified researcher.

## Author Contributions

BM and FS conceived the study. BM pursued the detailed computational research, performed the computations, verified the methods, and wrote the manuscript. MH developed the mathematical theory and provided corrections to the manuscript. FS supervised the findings of this work and provided corrections to the manuscript. TS supervised the HPX technical team. All authors discussed the results.

### Conflict of Interest Statement

The authors declare that the research was conducted in the absence of any commercial or financial relationships that could be construed as a potential conflict of interest.

## References

[B1] Blue Brain Project (2015). Coreneuron—Simulator Optimized for Large Scale Neural Network Simulations. Available online at: https://github.com/bluebrain/CoreNeuron

[B2] Brette R. Goodman D. F. (2011). Vectorized algorithms for spiking neural network simulation. Neural Comput. 23, 1503–1535. 10.1162/NECO_a_00123 21395437

[B3] Carnevale N. Rosenthal S. (1992). Kinetics of diffusion in a spherical cell. I. No solute buffering. J. Neurosci. Methods 41, 205–216. 10.1016/0165-0270(92)90086-S 1513181

[B4] Chindemi G. (2018). Towards a Unified Understanding of Synaptic Plasticity Parsimonious Modeling and Simulation of the Glutamatergic Synapse Life-Cycle. Lausanne: EPFL 10.5075/epfl-thesis-8186

[B5] Duran A. Ayguadé E. Badia R. M. Labarta J. Martinell L. Martorell X. (2011). Ompss: a proposal for programming heterogeneous multi-core architectures. Parallel Process. Lett. 21, 173–193. 10.1142/S0129626411000151

[B6] Gewaltig M. Diesmann M. (2007). NEST (NEural Simulation Tool). Scholarpedia 2:1430 10.4249/scholarpedia.1430

[B7] Goodman D. F. Brette R. (2008). The brian simulator. Front. Neurosci. 3:26. 10.3389/neuro.01.026.2009 20011141 PMC2751620

[B8] Hines M. (1984). Efficient computation of branched nerve equations. Int. J. Biomed. Comput. 15, 69–76. 10.1016/0020-7101(84)90008-4 6698635

[B9] Hines M. Kumar S. Schürmann F. (2011). Comparison of neuronal spike exchange methods on a blue gene/p supercomputer. Front. Comput. Neurosci. 5:49. 10.3389/fncom.2011.00049 22121345 PMC3219917

[B10] Hines M. L. Carnevale N. T. (1997). The neuron simulation environment. Neural Comput. 9, 1179–1209. 10.1162/neco.1997.9.6.1179 9248061

[B11] Hines M. L. Markram H. Schürmann F. (2008). Fully implicit parallel simulation of single neurons. J. Comput. Neurosci. 25, 439–448. 10.1007/s10827-008-0087-5 18379867 PMC2760991

[B12] Hodgkin A. L. Huxley A. F. (1952). A quantitative description of membrane current and its application to conduction and excitation in nerve. J. Physiol. 117, 500–544. 10.1113/jphysiol.1952.sp004764 12991237 PMC1392413

[B13] Kaiser H. Brodowicz M. Sterling T. (2009). Parallex an advanced parallel execution model for scaling-impaired applications, in International Conference on Parallel Processing Workshops, 2009. ICPPW'09 (Vienna: IEEE), 394–401.

[B14] Kale L. V. Krishnan S. Wilson G. V. Lu P. (1996). Parallel Programming using C++, in Charm++: Parallel Programming with Message-Driven Objects (MIT Press), 175–213.

[B15] Kandel E. R. Markram H. Matthews P. M. Yuste R. Koch C. (2013). Neuroscience thinks big (and collaboratively). Nat. Rev. Neurosci. 14, 659–664. 10.1038/nrn3578 23958663

[B16] Kissel E. Swany M. (2016). Photon: remote memory access middleware for high-performance runtime systems, in IEEE International Parallel and Distributed Processing Symposium Workshops (IPDPSW) (Chicago, IL: IEEE), 1736–1743.

[B17] Klijn W. Cumming B. Yates S. Karakasis V. Peyser A. (2017). Arbor: A morphologically detailed neural network simulator for modern high performance computer architectures, in 26th Computational Neuroscience Meeting (Antwerp).

[B18] Korf R. E. (1998). A complete anytime algorithm for number partitioning. Artif. Intell. 106, 181–203. 10.1016/S0004-3702(98)00086-1

[B19] Kozloski J. Wagner J. (2011). An ultrascalable solution to large-scale neural tissue simulation. Front. Neuroinform. 5, 10–3389. 10.3389/fninf.2011.00015 21954383 PMC3175572

[B20] Kulkarni A. Dalessandro L. Kissel E. Lumsdaine A. Sterling T. Swany M. (2016). Network-managed virtual global address space for message-driven runtimes, in Proceedings of the 25th ACM International Symposium on High-Performance Parallel and Distributed Computing (Kyoto: ACM), 15–18.

[B21] Kumar S. Heidelberger P. Chen D. Hines M. (2010). Optimization of applications with non-blocking neighborhood collectives via multisends on the blue gene/p supercomputer, in International Conference on Parallel and Distributed Processing Symposium, Vol. 2010 (Atlanta, GA: NIH Public Access), 1. 10.1109/IPDPS.2010.5470407 PMC3111918 21666880

[B22] Kumbhar P. Hines M. Fouriaux J. Ovcharenko A. King J. Delalondre F. (2019). CoreNEURON: an optimized compute engine for the NEURON simulator. arXiv preprint. arXiv:1901.10975. 10.3389/fninf.2019.00063 PMC6763692 31616273

[B23] Kumbhar P. Hines M. Ovcharenko A. Mallon D. A. King J. Sainz F. (2016). Leveraging a cluster-booster architecture for brain-scale simulations, in International Conference on High Performance Computing (Salt Lake City, UT: Springer), 363–380.

[B24] Magalhães B. R. C. Tauheed F. Heinis T. Ailamaki A. Schürmann F. (2016). An efficient parallel load-balancing framework for orthogonal decomposition of geometrical data, in International Conference on High Performance Computing (Frankfurt: Springer), 81–97.

[B25] Magalhães Bruno RC Blue Brain Project (2017). Neurox: A Parallel and Distributed Asynchronous Simulator of Extended Hodgkin-Huxley Neuron Models. Available online at: https://github.com/bluebrain/neurox

[B26] Markram H. Gerstner W. Sjöström P. J. (2012). Spike-timing-dependent plasticity: a comprehensive overview. Front. Synapt. Neurosci. 4:2. 10.3389/978-2-88919-043-0 22807913 PMC3395004

[B27] Markram H. Muller E. Ramaswamy S. Reimann M. W. Abdellah M. Sanchez C. A.. (2015). Reconstruction and simulation of neocortical microcircuitry. Cell 163, 456–492. 10.1016/j.cell.2015.09.029 26451489

[B28] Niebur E. (2008). Neuronal cable theory. Scholarpedia 3:2674 10.4249/scholarpedia.2674

[B29] Sodani A. (2015). Knights landing (KNL): 2nd generation Intel Xeon Phi processor, in IEEE Hot Chips 27 Symposium (HCS) (Cupertino, CA: IEEE), 1–24.

[B30] Sterling T. Anderson M. Bohan P. K. Brodowicz M. Kulkarni A. Zhang B. (2014). Towards exascale co-design in a runtime system, in Exascale Applications and Software Conference (Stockholm).

[B31] Vooturi D. T. Kothapalli K. Bhalla U. S. (2017). Parallelizing Hines matrix solver in neuron simulations on GPU, in IEEE 24th International Conference on High Performance Computing (HiPC) (Genoa: IEEE), 388–397.

[B32] Walker D. W. Dongarra J. J. (1996). MPI: a standard message passing interface. Supercomputer 12, 56–68.

[B33] Zenke F. Gerstner W. (2014). Limits to high-speed simulations of spiking neural networks using general-purpose computers. Front. Neuroinform. 8:76. 10.3389/fninf.2014.00076 25309418 PMC4160969

